# Hydroxyfasudil-Mediated Inhibition of ROCK1 and ROCK2 Improves Kidney Function in Rat Renal Acute Ischemia-Reperfusion Injury

**DOI:** 10.1371/journal.pone.0026419

**Published:** 2011-10-21

**Authors:** Dominik Kentrup, Stefan Reuter, Uta Schnöckel, Alexander Grabner, Bayram Edemir, Hermann Pavenstädt, Otmar Schober, Michael Schäfers, Eberhard Schlatter, Eckhart Büssemaker

**Affiliations:** 1 Department of Internal Medicine D, Experimental Nephrology, University of Münster, Münster, Germany; 2 Department of Nuclear Medicine, University of Münster, Münster, Germany; 3 European Institute for Molecular Imaging, University of Münster, Münster, Germany; 4 Dialysis Unit Hamm, Hamm, Germany; University of Colorado Denver, United States of America

## Abstract

Renal ischemia-reperfusion (IR) injury (IRI) is a common and important trigger of acute renal injury (AKI). It is inevitably linked to transplantation. Involving both, the innate and the adaptive immune response, IRI causes subsequent sterile inflammation. Attraction to and transmigration of immune cells into the interstitium is associated with increased vascular permeability and loss of endothelial and tubular epithelial cell integrity. Considering the important role of cytoskeletal reorganization, mainly regulated by RhoGTPases, in the development of IRI we hypothesized that a preventive, selective inhibition of the Rho effector Rho-associated coiled coil containing protein kinase (ROCK) by hydroxyfasudil may improve renal IRI outcome. Using an IRI-based animal model of AKI in male Sprague Dawley rats, animals treated with hydroxyfasudil showed reduced proteinuria and polyuria as well as increased urine osmolarity when compared with sham-treated animals. In addition, renal perfusion (as assessed by ^18^F-fluoride Positron Emission Tomography (PET)), creatinine- and urea-clearances improved significantly. Moreover, endothelial leakage and renal inflammation was significantly reduced as determined by histology, ^18^F-fluordesoxyglucose-microautoradiography, Evans Blue, and real-time PCR analysis. We conclude from our study that ROCK-inhibition by hydroxyfasudil significantly improves kidney function in a rat model of acute renal IRI and is therefore a potential new therapeutic option in humans.

## Introduction

AKI is a critical clinical condition associated with a high degree of morbidity and mortality despite best supportive care. However, at present, no effective treatment improving outcome is available. IRI is one of the main causes of AKI. It occurs in a broad spectrum of clinical settings including (transplantation) surgery, trauma, dehydration or sepsis leading to renal hypoperfusion, acute tubular necrosis (ATN), and functional disturbances - namely AKI. In renal transplantation it is a well known risk factor for delayed graft function, which prolongs hospitalization, increases costs, and needs a greater complexity of immunosuppressive drug management. Because IRI affects the kidney by reducing the number of nephrons and increases the risk of acute rejection episodes, it might cause a reduced graft survival.

Among the complex mechanisms involved in IRI, recent interest has focused on detailed studies of immune cells involved in the post-ischemic processes, thereby identifying inflammation as a key mediator of IRI. Even though the data regarding the cell types typically involved varies (e.g. due to the models used [1], [2] or due to non specific detection methods, e.g. myeloperoxidase, naphthol chloroacetate esterase, or HIS-48 staining [3]) it is well known that the increased influx of neutrophiles, T- and B-lymphocytes as well as macrophages/monocytes significantly contributes to the pathogenesis of AKI [4]. Neutrophiles and other attracted leukocytes adhere to endothelial cells using specific adhesion molecules such as intercellular adhesion molecule 1 (Icam1) and vascular cell adhesion molecule 1 (Vcam1) followed by transendothelial migration [5]–[9], often accompanied by plasma fluid and protein leakage [10]. In addition, activated leukocytes produce a variety of hyperpermeability factors, including cytokines, oxidants, proteases, lipid metabolites, and leukotrienes which directly or indirectly interact with the endothelium.

Recently, it has been shown that Rho effectors Rho-associated, coiled-coil containing protein kinases (ROCK) and their associated signaling pathways play pivotal roles in the development of (experimental) renal diseases [11]–[14]. ROCKs are protein serine/threonine kinases belonging to the AGC (PKA/PKG/PKC) family. They were the first effectors of Rho discovered [15]–[17]. To date, two ROCK isoforms, ROCK1 (ROKβ [18], p160ROCK [15]) and ROCK2 (ROKα [16], Rho kinase [17]) have been described. ROCKs are ubiquitously expressed [18], [19] and they phosphorylate various substrates [20]–[24]. Their involvement in the regulation of cellular motility, migration, adhesion, and transmigration is hereby of special interest. Notably, in leukocytes ROCKs are essential mediators for these processes [25]–[32]. Considering the important role of cytoskeletal reorganisation, mainly regulated by RhoGTPases, in the development of IRI-related AKI, we hypothesized that ROCK blockade may improve renal IRI outcome. Considering that leukocyte recruitment into the kidney is rather detrimental in the first stages of IRI, while providing possible beneficial effects in the later IRI stages, we chose a preventive approach using transitory ROCK-inhibition before and early in IRI [33].

Thus, using an animal model of renal IRI, we aimed to investigate whether ROCK-inhibition by hydroxyfasudil (HF), a specific inhibitor of ROCK1 and ROCK2 with an estimated half life of more than 5 hours, affects adhesion, migration, and transmigration of immune cells thereby reducing early, post IR leukocyte-endothelial interactions, endothelial leakage, post-ischemic inflammation, and kidney damage.

## Materials and Methods

### Animal models

Male Sprague Dawley (SD) rats (270–330 g, Charles River, Sulzfeld, Germany) with free access to food (standard rat chow, Altromin, Lage, Germany) and tap water were used. Experiments were approved by a governmental-committee on animal welfare (Landesamt für Natur, Umwelt und Verbraucherschutz Nordrhein-Westfalen, Permit # 8.87-50.10.36.09.020) and were performed in accordance with national animal protection guidelines. Surgeries were performed under anaesthesia with ketamine 100 mg/kg body weight (BW) intra peritoneal (i.p.) and xylazine 5 mg/kg BW i.p. (CEVA Tiergesundheit, Düsseldorf, Germany). Further doses of ketamine were injected as needed.

### IRI model

Before induction of IRI, rats underwent right nephrectomy. 7 days later, IRI was induced by clamping the left renal artery for 45 min (in a modification as published before [34]). Briefly, kidneys and vessels were approached through an abdominal midline incision. The left renal artery and vein were dissected free from connective tissue and from each other. All visible renal nerves were carefully detached from the vessels. Blood-flow through the left renal artery was interrupted by a vessel clip for warm ischemia. After 45 min of ischemia, the vessel clip was removed. The returning of original surface colour of the kidneys was confirmed visually, and the abdomen was closed in layers.

Rats were randomized into two groups: one hour before the ischemia procedure rats received either 10 mg/kg HF (i.p.) or vehicle solution (isotonic NaCl, i.p.). In the following reperfusion phase animals were housed for 1–4 days in metabolic cages; blood and urine samples were taken daily for analysis. On POD0, POD1 or POD4, respectively, kidneys were recovered for analyses.

**For assessment of renal perfusion by PET** IRI was induced as described above except of performance of the uninephrectomy. This variant was chosen because herein the native reference kidney and the contralateral kidney with IRI can be directly compared in the same animal.

### Blood-pressure

Mean systolic and diastolic blood-pressures were measured in conscious rats by use of a non-invasive, volume pressure recording technique (CODA, Kent Scientific Corporation, Connecticut) as published before [35]. First, animals were acclimatized to the system through daily training for 1 week prior uninephrectomy, followed by additional training after the animals had recovered. Final measurements were carried out immediately before the ischemia procedure (starting 30 min after the animals received either 10 mg/kg HF (i.p.) or vehicle solution (isotonic NaCl, i.p.) and ending 10 min before surgery). Each animal underwent a complete measurement cycle containing 25 consecutive single measurements from which means were calculated. All animals were trained by the same researcher at the same time of the day to minimize stress and the effect of biological rhythms.

### Image acquisition -PET

^18^F-FDG and ^18^F-fluoride were produced in a clinical routine setup on site using an RDS 111 cyclotron (CTI, Knoxville, TE, USA). PET scans were performed with the high-resolution multiwire chamber-based animal PET camera quadHIDAC (Oxford Positron Systems Ltd, Oxford, UK) [36]. Renal perfusion was assessed by ^18^F-fluoride PET. The perfusion index was calculated in a modification according to Hilson et al. as the ratio between the area under the arterial curve during the first 30 seconds*100 after tracer injection and the area under the renal curve [37].

^18^F-fluoride scans were performed at baseline, immediately after ischemia (POD0: briefly, the vascular clamp was recovered and the abdomen closed in layers with stitches. Thereafter, the operation tray with the rat was transferred to the PET. These procedures took approximately 10-15 min), and on POD4 from a dynamic whole body acquisition of 60 min length (to get additional information about renal fluoride clearance as a marker of renal function and about split renal function [34]) after tail vein injection of 15 MBq ^18^F-fluoride in 100 µl 0.9% NaCl. Thereafter, the catheter was purged with an additional 900 µl 0.9% NaCl solution. Acquisition started immediately after ^18^F-fluoride injection. During acquisition, rats were anaesthetized with oxygen/isoflurane inhalation (2% isoflurane, 0.7 l/min oxygen) and body temperature was maintained at physiological values by a heating pad.

### ^18^F-FDG-autoradiography

Autoradiography (*µ*-imager, Biospace Measures, Paris, France) was performed as published before [38], [39] on POD4. In short, 3 hours after i.v. injection of 30 MBq ^18^F-FDG in 100 µl 0.9% NaCl solution in a tail vein, kidneys were snap-frozen, sliced into 10 µm thick cryosections and activity was measured in a mid-coronary renal slice for 3 h.

### Evans Blue

Endothelial permeability was determined by assessing tissue concentration of Evans Blue (EB, Santa Cruz Biotechnology, Heidelberg, Germany). Complementary to the surgery animals received 30 mg/kg EB by tail vein injection at initiation of the reperfusion phase. After 30 min kidneys were perfused with 100 ml of isotonic NaCl, excised, shredded, and dehydrated. Samples were homogenized in formamide whereby total volume was adjusted to a 20x equivalent of sample dry weight, followed by 24 h incubation at 55°C. Supernatant was separated by centrifugation at 13.000 g for 30 min and EB concentration in the supernatant was quantified spectrophotometrically by measuring absorbances at 620 nm, as well as at 740 nm for the correction of contamination by heme pigments by applying a microplate reader (Infinite® F200, TECAN Deutschland GmbH, Crailsheim, Germany). Total concentration of EB was determined from generated EB standard curves absorbances, and expressed as µg/ml.

### Histology

Kidneys were recovered either on POD1 or POD4. The renal capsule was removed. Cross-sections were obtained, fixed in 4% buffered paraformaldehyde and embedded in paraffin. 5-µm-thick slices were then deparaffinized, rehydrated and stained with periodic acid Schiff (PAS). Digitalized pictures were taken with a microscope (Axiovert 100, Carl Zeiss AG, Oberkochen, Germany) equipped with a digital camera (Axiocam MRc, Carl Zeiss AG) using the AxionVisonLE Release 4.7.1 software (Carl Zeiss AG).

### Immunohistochemistry

Small pieces of kidney tissue were fixed in 4% formaldehyde in PBS for 12 h and embedded in paraffin. After deparaffinisation and rehydration with Clear Rite and descending ethanol series, 3-µm-thick sections were blocked with BSA 10% and immunostained employing the ABC method with antibodies against Mmp2 and Mmp9, respectively. Reaction products were visualized by DAB reaction. After counterstaining with haemalaun, images were acquired using an Axio Zeiss light microscope (Axiovert 100, Carl Zeiss AG, Oberkochen, Germany) equipped with a digital camera (Axiocam MRc, Carl Zeiss AG) using the AxionVisonLE Release 4.7.1 software (Carl Zeiss AG). Control stainings were performed without using primary antibody.

### Western Blotting Analysis

Kidneys of HF- and sham-treated animals were recovered 1 h after the animals had received their corresponding treatment. Subsequently, kidneys were lysed and submitted to immunoblotting analysis by using specific antibodies against phospho-MYPT1 (Thr853) (Cell Signaling) and GAPDH (Cell Signaling).

### Clinical Chemistry

Blood and urine samples were analyzed for electrolytes (ISE), creatinine (enzymatic assay; Creatinine-Pap, Roche Diagnostics, Mannheim, Germany) and blood urea nitrogen (BUN, urease-GLDH method) on a Roche Diagnostic analyzer (Modular P, Roche Diagnostics). Additionally, urine protein concentration (Bradford Blue; BioRad Laboratories, Germany) and osmolarity (Halbmikro-Osmometer, Knauer, Berlin, Germany) were measured.

### Gene expression analysis

Tissue samples were taken on POD1 or POD4, preserved in RNA*later* RNA Stabilisation Reagent (Qiagen, Hilden, Germany) and stored at −20°C. Gene expressions were analyzed by real-time PCR using the SYBR Green PCR Master Mix [40] (Applied Biosystems, Darmstadt, Germany) with the ABI PRISM 7900 Sequence Detection System. GAPDH was used as housekeeping gene. Relative gene expression values were evaluated with the 2-^ΔΔ^Ct method as described by Livak et al.. Primers are listed in table 1.

**Table 1:** Primer sequences used for gene expression analysis by real-time PCR and their corresponding genes.

Gene(official symbol & name)	Primer Sequence	
	Forward (5′-3′)	Reverse (5′-3′)
CD4 (CD4 molecule)	TGTGTCAGGTGCCGGCACCAACAG	GTGGGGCCCAGGCCTCATATG
CD8a (CD8a molecule)	AGGGAATGGGATTGGGCTTCGC	CTCTGAAGGTCTGGGCTTGAC
CD80 (CD80 molecule)	CGTTTGCCTGGGCAGGATCTG	GCTGCTTCCACAGGCCCTATG
CD86 (CD86 molecule)	GCTCTCAGATGCTGTTCCTGTG	ATAGTGTTCGTACAGAACCGAC
Ceacam1 (CD66a, carcinoembryonic antigen-related cell adhesion molecule 1)	GTGAAGCCCGGAACCCAGCG	GTCTGCATGGCAGGAGAGGTTG
Fcnb (ficolin B)	GAACCAATGAGCTGCGGGTGG	GTTTTGGGAAGTCAGGGAGTCAC
Foxp3 (forkhead box P3)	GTGGTGCAGTCTCTGGAGCAGC	CAGGAGCTCTTGTCCACTGAGGC
Gapdh (glyceraldehyde-3-phosphate dehydrogenase)	CATCAACGACCCCTTCATT	ACTCCACGACATACTCAGCAC
Icam1 (intercellular adhesion molecule 1)	CGGGAGATGAATGGTACC	GCGGTAATAGGTGTAAATGG
Ifng (interferon gamma)	GTCATCGAATCGCACCTGATC	GGCTAGATTCTGGTGACAGCTG
Il2ra (CD25, interleukin 2 receptor, alpha)	GCAGTGGCCAGCTGCATCTTC	CTAGCTTGCTAGATGGTTCTTCTGC
Kim1 (Havcr1, kidney injury molecule1)	GAGCACCGTGGTTGTCACCAGG	GTAGATGTTGTCTTCAGCTCGGG
Mmp2 (matrix metalloproteinase 2)	GCTCAGATCCGTGGTGAGATCTTC	TTTCCGGGAGCTCAGGCCAGAATG
Mmp7 (matrix metalloproteinase 7)	GCCACTCATGAACTTGGCCAC	CTGCATCTATCACAGCTTGTTCC
Mmp8 (matrix metalloproteinase 8)	ATCTGGAGTGTGCCATCAACCC	CCGGCCTGGTTGAAAGGCATG
Mmp9 (matrix metalloproteinase 9)	AAGGCCATTCGTTCACCGCGC	CACGTCTCGCGGCAAGTCTTC
Ms4a1 (CD20, membrane-spanning 4-domains, subfamily A, member 1)	CTGTGGGGAGGCATTATGTAC	CCAGAAATGGCAGCAAAGAGGC
Ncam1 (CD56, neural cell adhesion molecule 1)	CTGACATGTGAAGCCTCCGGAG	CTCTTGCTTCTCTGGTCGAGTC
Tnf (tumor necrosis factor)	AAGTTCCCAAATGGGCTCCCTC	GCTCCTCCGCTTGGTGGTTTG
Tnfsf15 (tumor necrosis factor (ligand) superfamily, member 15)	GTGACAGAAGAGAGGTCTGCC	GGTTCTTGGTGAAGGCCATCC
Vcam1 (vascular cell adhesion molecule 1)	CTGTTTGCAGTCTCTCAAGC	GCTTCAAAGCCTTCTTTGTGC

### Statistics

Statistical analyses were performed using GraphPad Prism version 4.0 (GraphPad Sofware, La Jolla, CA, USA). Values are expressed as mean ± SEM. Comparison among groups was performed by one-way ANOVA along with post-hoc Tukey test, whereas a significance level of p<0.05 was defined as statistically significant.

## Results

### ROCK-inhibition improves post-ischemic renal function

**Figure 1:**
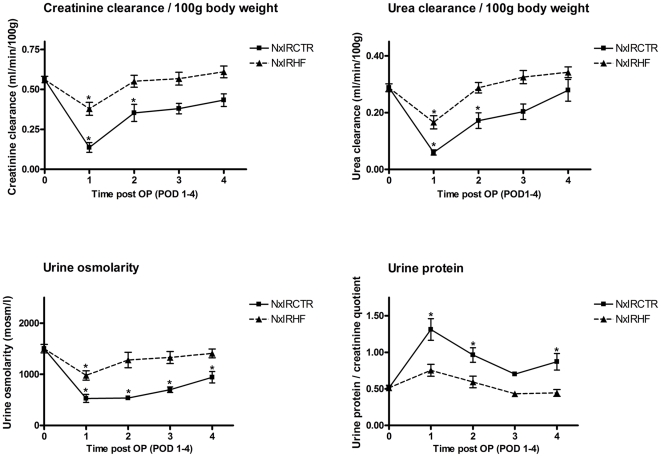
Effects of ischemia-reperfusion injury and ROCK-inhibition on metabolic parameters. IRI significantly affected kidney function (as assessed by creatinine-clearance, urea-clearance, urine osmolarity and urine protein excretion on POD0-4) whereas the renal function of HF-treated animals was less severe affected. Starting on POD 1, HF-treated animals had improved creatinine-clearance (NxIRHF: 0.38±0.04 ml/min/100 g vs. NxIRCTR: 0.14±0.03 ml/min/100 g, n = 6) as well as urea-clearance (NxIRHF: 0.17±0.02 ml/min/100 g vs. NxIRCTR: 0.06±0.01 ml/min/100 g, n = 6) when compared to vehicle-treated rats. Moreover, the HF-treated rats showed a better preserved renal urinary concentrating capacity. Interestingly, proteinuria, which initially occurred in the NxIRCTR group was absent in NxIRHF. In summary, kidney injury was significantly attenuated by HF application leading to a distinct faster recovery of renal function. Values are expressed as mean ± SEM; *indicates significance to NxIRCTR POD0.

To estimate renal function, blood and urine samples were analyzed at baseline, as well as after IRI on POD1 and 4. Before starting the interventions, parameters did not differ between groups (Table 2). One week after uninephrectomy no significant differences to healthy animals were observed. Due to the IRI renal function significantly decreased in all intervention groups by POD1. Recovery of renal function started immediately thereafter leading to a continuous decrease of Cr_S_ and BUN until POD4. However, HF-treated rats showed a significantly improved creatinine-clearance as well as urea-clearance. Furthermore, urine osmolarity, proteinuria, and polyuria improved significantly as shown in figure 1 and table 2. To sum up, renal damage was reduced and kidney function of HF-treated animals recovered faster than in controls. While kidney function normalized already on POD2 in NxIRHF (no significant differences compared to baseline values), kidney function of untreated rats failed to normalize even 48 h later at least for some parameters.

**Table 2:** Effects of IRI on functional parameters of either vehicle-treated (NxIRCTR) or hydroxyfasudil-treated (NxIRHF) animals.

	POD-7	POD0	POD1		POD2		POD3		POD4	
	NxIRCTR&NxIRHF(n = 14)	NxIRCTR&NxIRHF(n = 18)	NxIRCTR(n = 8)	NxIRHF(n = 10)	NxIRCTR(n = 6)	NxIRHF(n = 8)	NxIRCTR(n = 6)	NxIRHF(n = 8)	NxIRCTR(n = 8)	NxIRHF(n = 10)
**Urine volume** **(ml/24 h)**	14.08±0.93	17.64±0.88	26.95±2.82*	18.92±1.72	43.6±2.85*	22.03±2.20	30.86±2.16*	18.92±1.24	29.47±2.26*	19.58±0.97
**Urine osmolarity** **(mmosm/kg)**	1909±120.8	1502±82.01	527.5±78.13*	978±92.30*	536.8±27.13*	1280±150.0	698.8±53.29*	1328±117.9	941±111.1*	1409±86.30
**Na^+^ in serum** **(mM)**	142.8±1.02	147.7±1.28	146±2.29	146.2±1.19	147.7±0.92	145.3±1.37	146.7±0.95	145.8±1.42	146.3±2.89	143.1±2.14
**Na^+^ in urine** **(mM)**	102±6.57	84.67±6.33	17.88±3.1*	32.5±8.26*	28±3.01*	71.13±11.64	33.67±5.39*	62.13±8.62	56.88±9.25	91.3±5.97
**FE_Na+_** **(%)**	0.22±0.01	0.38±0.03	0.52±0.18	0.23±0.04	0.62±0.11	0.40±0.06	0.44±0.05	0.32±0.07	0.58±0.06	0.44±0.02
**K^+^ in serum** **(mM)**	4.99±0.18	4.89±0.1	6.35±0.58	5.44±0.52	5.1±0.18	5.23±0.16	4.92±0.22	5.05±0.33	4.94±0.12	4.82±0.22
**K^+^ in urine** **(mM)**	276.9±15.31	255.6±12.93	97.45±8.25*	131.5±16.14*	83.67±5.67*	174.7±22.36*	102.8±10.72*	203.3±18.95	149.6±20.96*	225.2±12.76
**FE_K+_** **(%)**	17.25±0.95	35.74±2.61	80.8±22.95*	26.94±2.75	52.12±11.69	25.91±1.82	41.17±2.63	29.62±3.30	45.8±2.10	32.94±1.65
**Proteine excretion** **(mg/mg creatinine)**	0.31±0.02	0.51±0.03	1.31±0.15*	0.75±0.08	0.96±0.1*	0.59±0.08	0.70±0.03	0.43±0.02	0.87±0.11*	0.44±0.05
**Serum creatinine** **(Cr, mg/dl)**	0.19±0.01	0.35±0.01	1.10±0.19*	0.52±0.06	0.68±0.10*	0.38±0.03	0.51±0.07	0.36±0.03	0.44±0.05	0.34±0.02
**Blood urea nitrogen** **(BUN, mg/dl)**	17.93±0.62	24.17±0.84	62±8.93*	35.2±3.96	53.17±6.96*	27±1.39	43±7.08*	23.25±1.21	36.14±6.46	23±1
**CrCl** **(ml/min/100 g BW)**	0.97±0.05	0.56±0.02	0.14±0.03*	0.38±0.04*	0.35±0.05*	0.55±0.04	0.38±0.03	0.57±0.04	0.43±0.04	0.61±0.04
**BUN-Cl** **(ml/min/100 g BW)**	0.39±0.02	0.29±0.01	0.06±0.01*	0.17±0.02*	0.17±0.03*	0.29±0.02	0.20±0.03	0.33±0.02	0.28±0.04	0.34±0.02

Mean values ± SEM with the number of animals in parentheses;

*p<0.05 vs. POD0. BW: body weight, POD: postoperative day, FE_Na+_: fractional excretion of Na^+^, FE_K+_: fractional excretion of K^+^.

### Effects of HF on histological changes and on ^18^F-FDG-uptake in autoradiography

**Figure 2:**
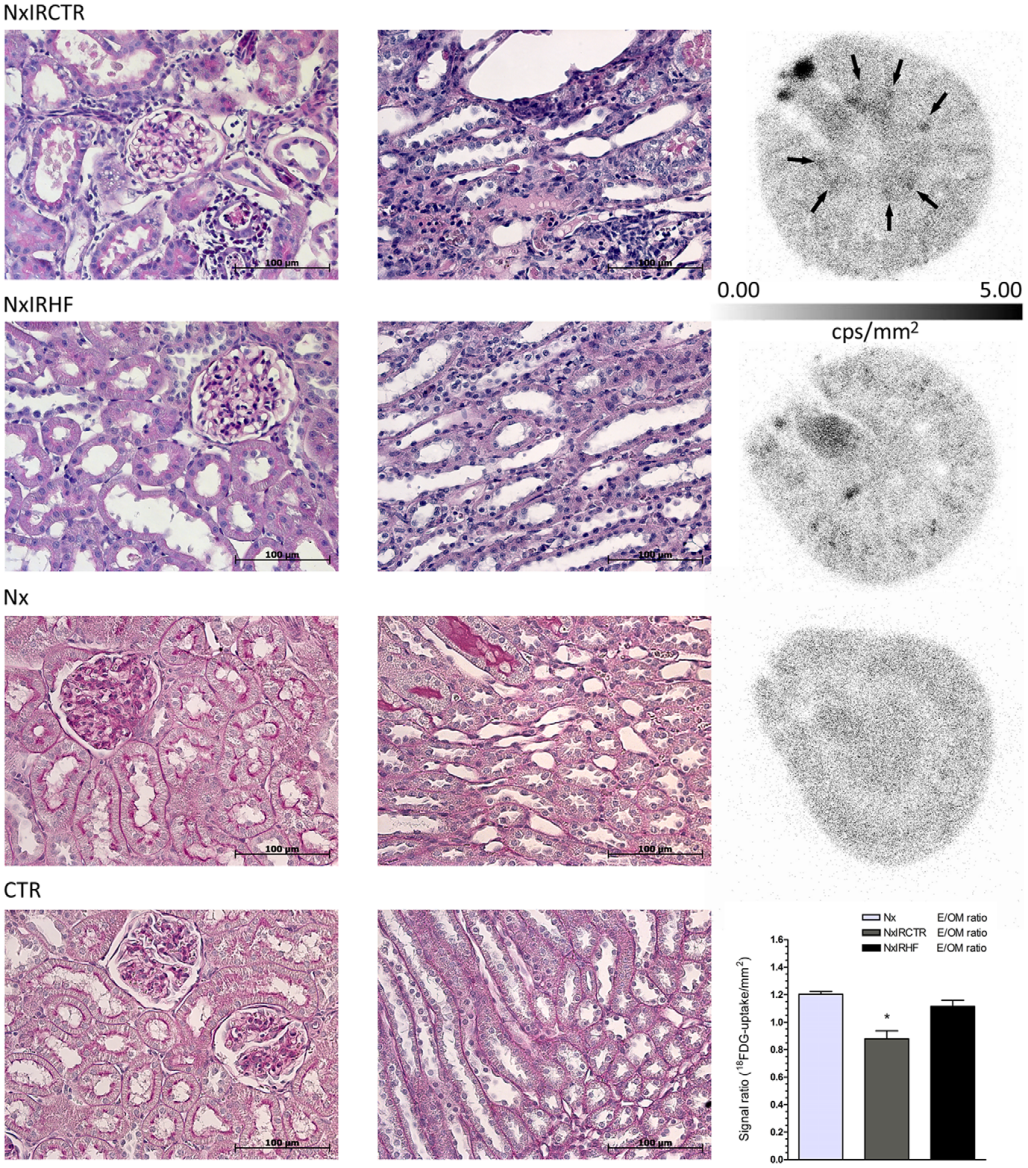
Effect of ischemia-reperfusion injury and ROCK-inhibition on histological changes. Shown are representative PAS-stainings (cortex and medulla) and ^18^F-FDG-autoradiographies of post-ischemic kidneys on POD4 (NxIRCTR, NxIRHF), as well as PAS-stainings of kidneys from healthy (CTR) and uninephrectomized animals (Nx). Following IRI kidneys presented with signs of ATN, i.e. tubular dilation, swelling and necrosis in addition to intraluminal brush border debris and protein casts. Leukocyte infiltration was moderate and emphasized in the outer medulla. Kidneys of HF-treated animals (NxIRHF) showed reduced signs of ATN including attenuated interstitial infiltration when compared with vehicle-treated animals (NxIRCTR). In congruence, we found distinct ^18^F-FDG-uptake (quantified as counts per second (cps)/mm^2^) in the outer medulla region of NxIRCTR (arrows in autoradiography) with ATN whereas this was absent in the NxIRHF group. This was statistically verified by calculating the ratio (E/OM), between the ^18^F-FDG-uptake in the entire organ slice (E) compared to that in the outer medulla (OM). While there is no statistical difference between uninephrectomized (Nx: 1.2±0.02) and ROCK-inhibitor treated (NxIRHF: 1.12±0.04) animals, the E/OM ratio of untreated ischemic animals (NxIRCTR: 0.88±0.06) is significantly different from both aforementioned. Values are expressed as mean ± SEM, n = 3. * p<0.05 vs. Nx & NxIRHF). Non-marked, intensively stained areas are artifacts due to renal ^18^F-FDG excretion.

To estimate renal damage and infiltration (also for validation of ^18^F-FDG data) we evaluated renal histology (Figure 2) in addition to metabolic parameters. Subsequent to IRI (POD4), kidneys presented with signs of ATN, i.e. tubular dilation, swelling, and necrosis, in addition to intraluminal brush border debris and protein casts. Leukocyte infiltration was moderate and emphasized in the outer medulla. Corresponding to improved renal function, kidneys of NxIRHF showed reduced signs of ATN including attenuated interstitial infiltration when compared with NxIRCTR. Examination of inflamed tissue by autoradiography has shown that ^18^F-FDG-accumulation is correlated to the degree of inflammatory infiltration in different pathophysiological scenarios [38], [41]. Thus, we amended histological evaluation by autoradiographic assessment of mid-coronary kidney slices in order to visualize inflamed tissue areas. Exemplary measures are given in figure 2. In congruence to histological data, we found a distinct ^18^F-FDG-uptake in the outer medulla region of NxIRCTR with ATN whereas this was absent in NxIRHF. Both, histologic and autoradiographic evaluation of kidneys showed distinct amelioration of renal damage (less structural damage, less inflammation) in the HF-treated group.

### Effects of HF on ROCK-activity, renal perfusion and blood-pressure

**Figure 3:**
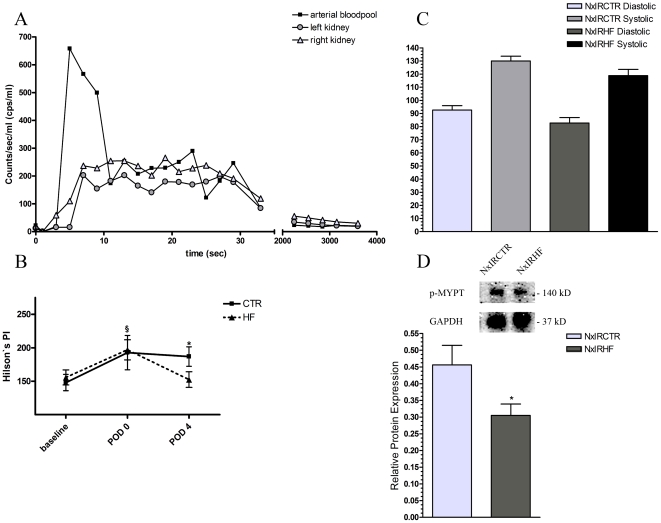
Effect of ischemia-reperfusion injury and ROCK-inhibition on renal perfusion. A) Exemplary, representative time activity curve (TAC) of the arterial bloodpool and of both kidneys in a control rat on POD4. Areas under the curves during the first 30 seconds after ^18^F-fluoride injection were used to calculate the perfusion index (perfusion index left kidney: 310.7 (post IR), right kidney: 185.6 (CTR)). B) Comparison of the modified Hilson's Perfusion index between the two groups as assessed by ^18^F-PET. At the beginning of the reperfusion phase (POD0, post-ischemia) kidney perfusion was significantly impaired in both groups (CTR POD0: 192.8±25.7, n = 7 and HF POD0: 197.0±15.0, n = 8) compared with baseline values (CTR: 148.0±12.3, n = 9 vs. HF: 155.7±11.2, n = 9). However, kidney perfusion of the HF-treated rats has already recovered on POD4 while perfusion of the vehicle-treated group was still impaired (CTR POD4: 186.9±14.5, n = 6 vs. HF POD4: 152.4±11.6, n = 8) (§ p< 0.05 vs. baseline,* p<0.05 HF POD4 vs. CTR POD4). C) Mean systolic and diastolic blood-pressures of conscious rats before ischemia induction on POD0. No significant differences were detected between HF- and sham-treated animals (NxIRCTR 130.0±3.6 mmHg (systolic) 92.6±3.3 mmHg (diastolic), n = 8 vs. NxIRHF 118.8±4.8 (systolic) 82.7±4.2 (diastolic) n = 7, p<0.05). D) Relative phospho-MYPT1 expression in relation to GAPDH for HF- and sham treated animals as assessed by quantitative Western Blot analysis (NxIRCTR 0.46±0.06 vs. NxIRHF 0.31±0.03, n = 7, p<0.05).

ROCK-inhibition was verified by evaluating the phosphorylation status of the ROCK-substrate phospho-MYPT1 by quantitative Western Blot analysis. As depicted in Figure 3D, in NxIRHF ROCK-activity was significantly reduced in kidney samples taken immediately before the ischemia procedure (NxIRCTR 0.46±0.06 vs. NxIRHF 0.31±0.03, n = 7, p<0.05). Additionally, even though our results indicated a crucial role of ROCK-dependent leukocyte recruitment in renal IRI so far, the clinical field of application of ROCK-inhibitors (e.g., Fasudil) lies within cardiovascular diseases, where mainly vasodilatatory and therefore blood-pressure lowering effects are observed. Hence, one might assume that the post-ischemic functional improvement of kidneys was mediated by increased perfusion (via vasodilatation). To test this hypothesis, we assessed renal perfusion by ^18^F-PET and calculated a modified Hilson's Perfusion index (Hilson's PI, Figure 3, higher values indicate lower perfusion rates). At baseline renal perfusion did not differ between groups and kidneys (CTR: 148.0±12.3, n = 9 vs. HF: 155.7±11.2, n = 9). Ischemia led to a significant decrease of renal perfusion in the affected kidney only as assessed immediately after the ischemia inducing operation. Again, there was no difference between NxIRCTR POD0 (192.8±25.7, n = 7) and NxIRHF POD0 (197.0±15.0, n = 8). However, on POD4 renal perfusion in vehicle-treated animals was still impaired while the renal perfusion of HF-treated rats had completely recovered (NxIRCTR POD4: 186.9±14.5, n = 6 vs. NxIRHF POD4: 152.4±11.6, n = 8). In addition, blood-pressure lowering effects of HF were excluded by use of a non-invasive, volume pressure recording technique (Figure 3C) which is consistent with work published by Komers et al. [42].

### Effect of ROCK-inhibition on renal endothelial permeability after IRI

**Figure 4:**
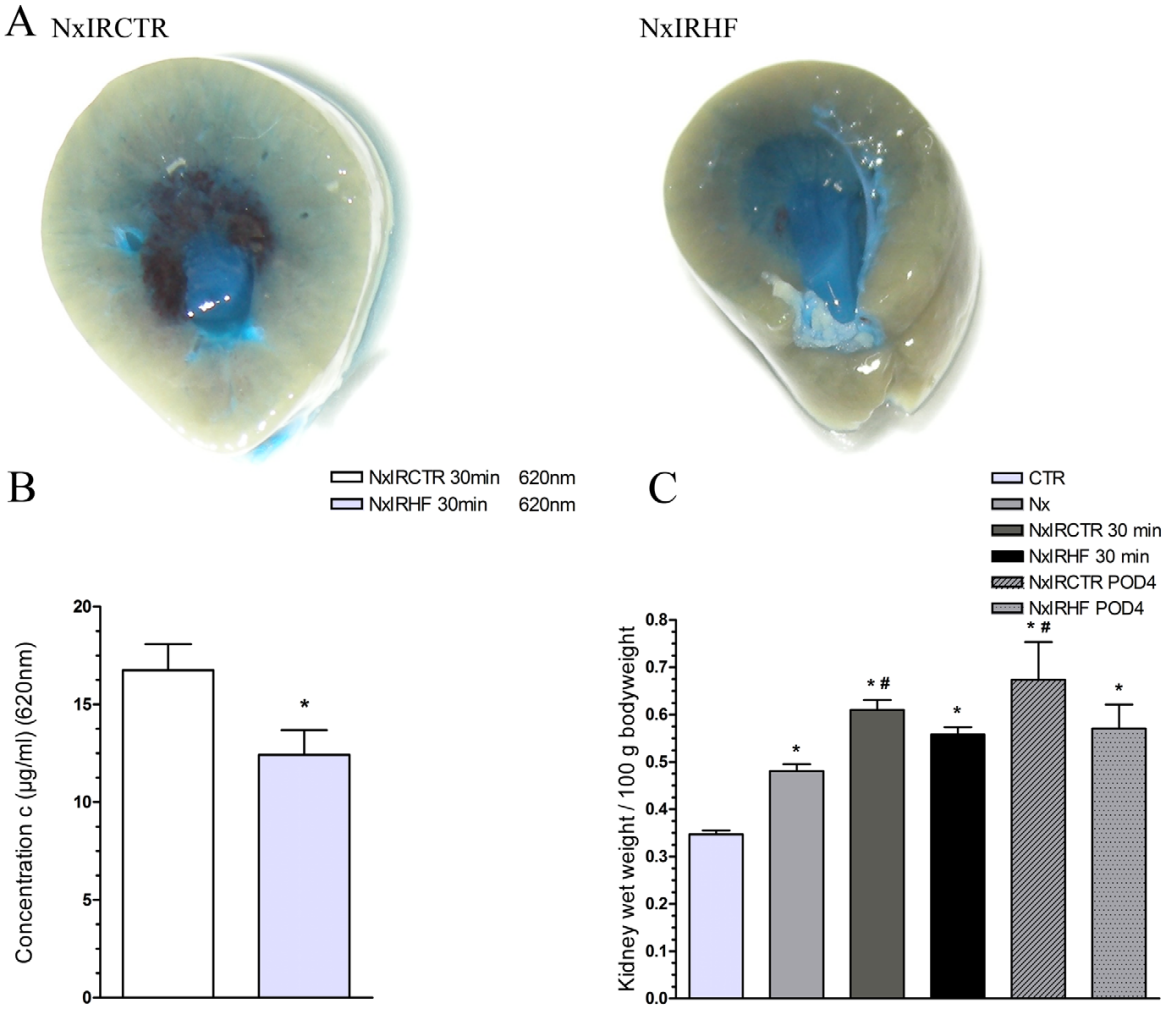
Effect of ischemia-reperfusion injury and ROCK-inhibition on renal vascular permeability. A) Renal vascular permeability as assessed by Evans Blue (EB) tissue accrual after 30 min of reperfusion in renal sections. Kidney of untreated (NxIRCTR) rats exhibit thereby increased accumulation of EB compared with HF-treated rats (NxIRHF) as assessed by B) spectrophotometrical quantification at 620 nm (NxIRCTR: 16.7±1.3 µg/ml, n = 4, NxIRHF: 12.4±1,3 µg/ml, n = 5) (* p<0.05 vs. NxIRCTR). C) Evaluation of kidney wet weight/100 g body weight ratio. In comparison with healthy animals (CTR) kidney weight significantly increased in all groups. However, compared with uninephrectomized animals (Nx) the kidney wet weight/100 g body weight ratio only increased significantly in untreated ischemic animals (NxIRCTR 30 min & POD4), while there was no significant increase in the ROCK-inhibitor treated group (CTR: n = 18, Nx: n = 6, NxIRCTR 30 min: n = 7, NxIRHF 30 min: n = 8, NxIRCTR POD4: n = 3, NxIRHF POD4: n = 3; *  =  p<0,05 vs. CTR, # p<0,05 vs. Nx).

Our results so far indicated a HF-related modulation of the inflammatory response affecting adhesive and migratory capabilities of immune cells. Additionally, HF-treatment modifies adhesion molecule expression and renal perfusion in the long run, which indicates HF-effects on endothelia. Thus, we aimed to test endothelial functionality by applying an Evans Blue (EB)-based vascular permeability assay during the initial 30 minutes of the reperfusion phase (Figure 4). Performing spectrophotometrical analyses of the renal content of EB after IRI, we assessed a distinct higher accumulation of EB in untreated (NxIRCTR: 16.7±1.3 µg/ml, n = 4) than in HF-treated rats (NxIRHF: 12.4±1.3 µg/ml, n = 5) (* p<0.05 vs. NxIRCTR).

### Effects of HF on the expression of matrix metalloproteinases

**Figure 5:**
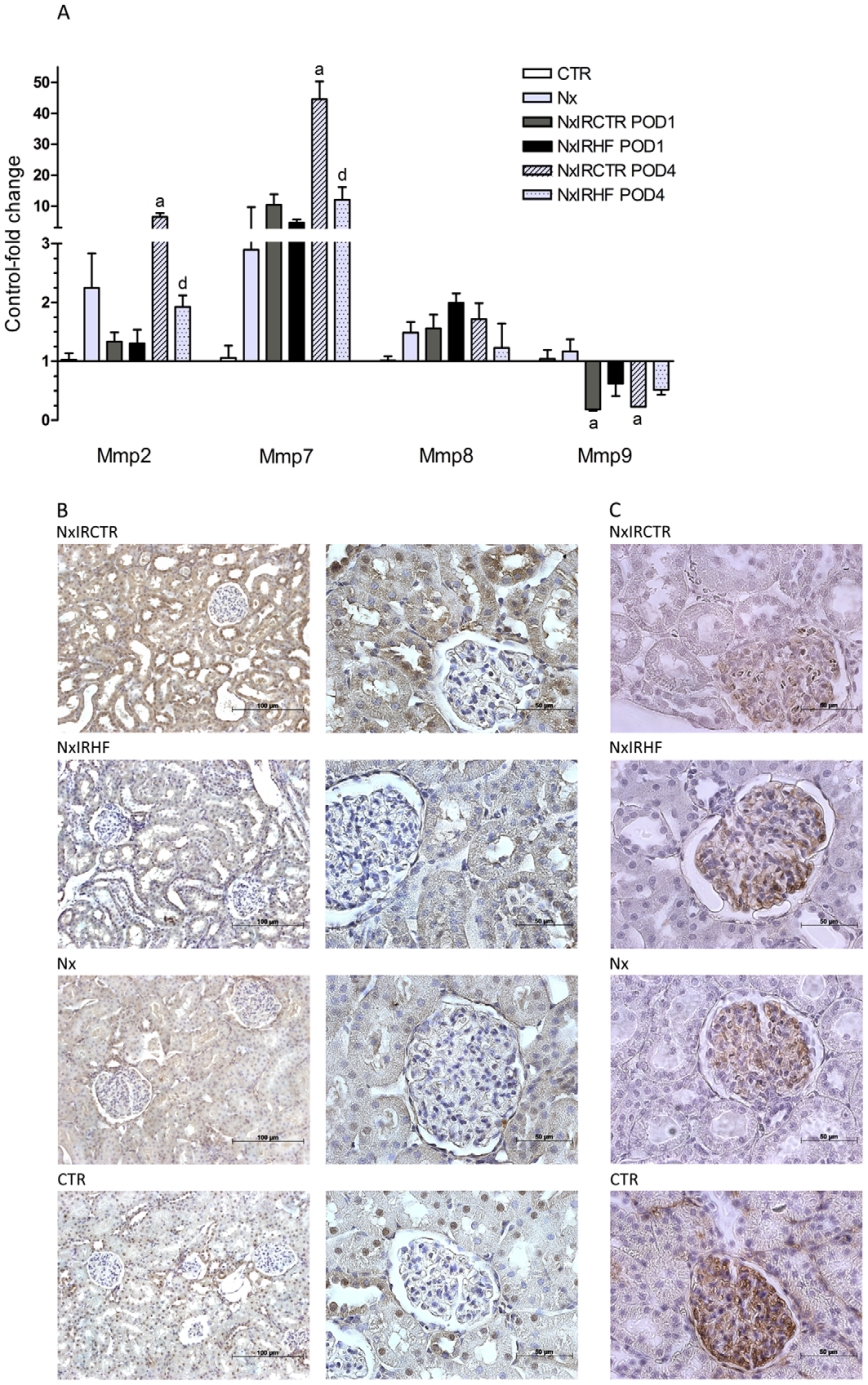
Effect of ischemia-reperfusion injury and ROCK-inhibition on matrix metalloproteinases expression. A) The mRNA-expression of Mmp2, Mmp7, Mmp8 and Mmp9 in whole kidney lysates as analyzed by real-time PCR. In vehicle-treated rats ischemia-reperfusion injury led to a significantly increased mRNA-expression of Mmp2 and Mmp7 on POD4, as well as decreased mRNA-expression of Mmp9 on POD1 and POD4 when compared to healthy and HF-treated animals. In turn, mRNA-expression of Mmp2 and Mmp7 of HF-treated animals did not change significantly compared to healthy animals. Differences in gene-expression were reflected by corresponding protein-expression as shown by immunohistochemistry of Mmp2 (B) and Mmp9 (C). Values are expressed as mean ± SEM, n = 4–5. **a** p<0.05 vs. CTR, **b** p<0.05 vs. NxIRCTR POD1, **c** p<0.05 vs. CTR and NxIRCTR POD1, **d** p<0.05 vs. NxIRCTR POD4, **e** p<0.05 vs. CTR and NxIRCTR POD4.

In the next step, we aimed to clarify if the infiltrates observed after IRI were harmful to the kidney. Therefore, we employed real-time polymerase chain reaction (PCR) and immunohistochemical analyses of matrix metalloproteinases (Mmp). The mRNA-expression results of Mmp2, Mmp7, Mmp8, and Mmp9 are presented in figure 5A. Whereas upon IRI-treatment, vehicle-treated animals demonstrated up-regulated levels of Mmp2 and Mmp7, Mmp9 was down-regulated when compared to HF-treated animals. Exemplary immunohistochemistry of Mmp2 and Mmp9 confirmed these findings also on the protein-expression level (Figure 5B and C).

### Effects of HF on mRNA-expression analyses of selected genes

**Figure 6:**
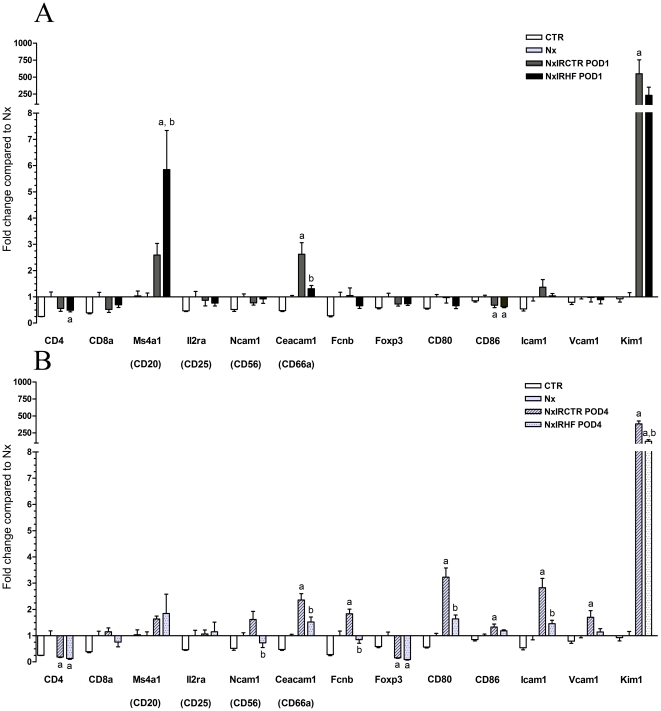
Effect of ischemia-reperfusion injury and ROCK-inhibition on mRNA-expression. Fold change in mRNA-expression compared to uninephrectomized animals (whole kidney lysates) as analyzed by real-time PCR (CD4, CD8a, CD20/Ms4a1, CD25/Il2ra, CD56/Ncam1, CD66a/Ceacam1, Fcnb, Foxp3, CD80, CD86, Kim1, Icam1, Vcam1) on POD1 (A) and POD4 (B). Following IRI, we observed a distinct up-regulation of marker mRNA, B-cells (CD20), neutrophil granulocytes (CD66a), activated B-cells/monocytes (CD80), antigen presenting cells (CD86) and the monocyte/macrophage activity marker ficolin B (Fcnb). IRI also led to a distinct up-regulation of mRNA-levels of vascular adhesion molecules (Icam1, Vcam1) and, especially, of Kim1. ROCK-inhibition significantly attenuated the up-regulation of CD66a, CD80, CD86, FcnB, Icam1, Vcam1 and Kim1. CD86 and Vcam1 were not significantly different in NxIRCTR and NxIRHF. Values are expressed as mean ± SEM, n = 4–6. **a** p<0.05 vs. Nx, **b** p<0.05 vs. NxIRCTR POD1 or NxIRCTR POD4, respectively.

Because ROCK-inhibition considerably improved renal recovery after IRI and reduced inflammation, we aimed to analyze the expression of possibly involved genes. Although earlier analyses have linked ROCK-inhibition to reduced infiltration of macrophages into the (post-ischemic) kidney [11], [12], [43] a precise analysis of the immune cell types, i.e. neutrophiles, monocytes, as well as B- and T-cells, involved is lacking. Recently, it has been shown that ROCK-inhibition generally negatively influences the ability of leukocytes to adhere and to (trans-) migrate [25], [26], [28], [30], [32]. Moreover, ROCK-inhibition significantly influences the integrity and permeability of endo- and epithelia. In a first step, we analyzed marker genes of specific leukocyte subpopulations on POD1 and POD4 (Figure 6) to characterize the post-ischemic infiltrate and the effect of HF on different immune cell types. On POD1 following IRI, there was no significant difference between the HF treated and untreated IRI rats, besides the mRNA of the up-regulated B-cell marker CD20 and the neutrophil granulocytes marker Ceacam1, which was downregulated in the HF group. On POD4, HF treatment attenuated the up-regulation of Ceacam1, CD80 (activated B-cells/monocytes), CD86 (antigen presenting cells and the monocyte/macrophage activity marker ficolin B. As a second gene set, we measured mRNA-expression of pro-inflammatory cytokines such as interferon gamma (Infg), tumor necrosis factor (Tnf), tumor necrosis factor (ligand) superfamily member 15 (Tnfsf15), adhesion molecules (Icam1, Vcam1), and kidney injury molecule 1 (Kim1). On POD1, we assessed a distinct up-regulation of Kim1 only, whereas on POD4 Icam1, Vcam1, and Kim1 were up-regulated. HF, treatment attenuated the up-regulation of Kim1 (POD1 and 4), Icam1 and Vcam1 (POD4). However, in the latter case (Vcam1) expression was only attenuated in NxIRHF when compared to uninephrectomized animals but failed to be significantly downregulated when compared to NxIRCTR. Ifng, Tnf and Tnfsf15 were not significantly different expressed on POD1 and 4; Figure 6 and Figure S1 (for Figure S1 see supplemental data)).

## Discussion

Considering the important role of RhoGTPases in cytoskeletal reorganization of endothelia and leukocyte migration, we hypothesized that preventive ROCK-inhibition may improve renal IRI outcome by reducing cellular infiltration. To test our hypothesis and to elucidate the mechanisms involved, we used an animal model of IRI. Thereby, we were able to show that renal IRI was substantially reduced in animals which received the ROCK-inhibitor HF. Renal function recovered earlier and inflammation was distinctly lower in HF-treated rats suggesting a critical role of ROCK in the development of (renal) IRI. We herein show that several mechanisms contributed to the observed ROCK-dependent IRI changes, including changes in the expression of endothelial adhesion molecules, matrix metalloproteinases and increased endothelial leakage/vascular permeability accompanied by leukocyte recruitment and transmigration.

Reviewing the literature this is the first *in vivo* study showing that HF-mediated ROCK-inhibition not only improves IRI-induced tubular damage as illustrated on the cellular and subcellular level but that it also improves kidney function (former studies used other ROCK-inhibitors [43]–[46]). Phenotypical implications of ROCK-inhibition include improved renal function and reduced leukocyte recruitment into the post-ischemic kidney. Morphological signs of IRI in kidneys of untreated animals included signs of severe acute tubular necrosis such as tubular dilation, swelling, and necrosis in addition to intra luminal brush border debris and protein casts. Leukocyte infiltration was moderate and emphasized in the outer medulla. This corresponded with an increased accumulation of ^18^F-FDG, as assessed by autoradiography, indicating elevated metabolic rates due to inflammatory infiltration in these areas [38], [41]. In addition, mRNA-expression levels of Mmp2 and Mmp7, which are major regulators of extracellular matrix degradation/turnover, and are therefore critical for tissue homeostasis [47] increased after IRI in kidneys of untreated animals, while mRNA-expression of Mmp9 decreased. Changes in mRNA-levels were reflected by corresponding alterations in protein-expressions (e.g. Mmp2 and Mmp9). Both, increased expressions of Mmp2 as well as decreased expression of Mmp9 have been described to promote kidney injury [48], [49]. Consistent with the relation of mRNA-expression of Mmp2 and Mmp7 to the degree of infiltrating cells (activated leukocytes produce high amounts of Mmps [50]), levels of Mmp2 and 7 increased in kidneys of untreated animals which underwent IRI and presented inflammatory infiltration. ROCK-inhibition by HF negated both, increased expression of Mmp2 and Mmp7, as well as downregulation of Mmp9. This hints towards a lower degree of IR-related tissue damage/inflammation in HF-treated animals. Among others, these results indicated that leukocytes might play a crucial role in how ROCK-inhibition affects the development of IRI, especially in the early stages. Interestingly, it has been shown in mice that knockout of ROCK1 significantly reduces leukocyte recruitment and neointima formation following vascular injury [51]. However, even though some studies claim that kidney infiltration with macrophages seems to be decreased by ROCK-inhibition [11], [12], [43], a comprehensive analysis of immune cell types involved is lacking. Thus, we used RT-PCR analysis to confirm and characterize the pattern of inflammatory cells and to identify ROCK-dependent changes therein. By analyses of marker genes of leukocyte subpopulations, we observed in untreated post-ischemic kidneys a distinct up-regulation of marker mRNA of B-cells (CD20), neutrophil granulocytes (CD66a), activated B-cells/monocytes (CD80/B7-1), antigen presenting cells (CD86) and the monocyte/macrophage activity marker ficolin B (Fcnb). While CD66a and CD20 were already up-regulated on POD1, we detected increased expression of the other marker genes only on POD4. This is in congruence to Li et al. who showed that neutrophils are the spearhead cell type in IRI [52], [53]. However, some IRI studies performed with neutrophil-depleted animals failed to show a protective effect on renal IRI compared with controls [1], [54]. Similarly, it is well known that IRI occurs also in neutropenic patients [55]. Thus, leukocytes others than neutrophils seem to be involved as well. Therefore, among others Takada et al. stated that mononuclear leukocytes may be the main effector cells involved in IRI [56], [57]. Nevertheless, ROCK-inhibition significantly prevented an up-regulation of all mentioned marker genes including neutrophils, B7-1, and mononuclear cells in kidney tissue following IR. These findings indicate that ROCK-activity substantially participates in IRI via modulation of leukocyte infiltration. Consistent with this, it was shown that ROCK is important for migration of leukocytes [25]–[32]. The importance of cell migration for inflammatory responses was supported recently [33]. Inhibition of the motor protein non-muscle myosin II which impairs the actinomyosin powered locomotive machinery improves the progression of experimental obstructive nephropathy which serves as a model of progressive renal disease mainly by its potent anti-inflammatory effect [58]. In a model of hypertensive nephropathy inhibition of lymphocyte migration limited histological and molecular fibrosis without affecting increased systemic blood-pressure [59]. As immune cell migration depends on endothelial adhesion molecules and as neutrophils are capable of producing a variety of hyperpermeability factors, e.g. cytokines which directly or indirectly interact with the endothelium, we analyzed a second gene set including pro-inflammatory cytokines (Ifng, Tnf, Tnfsf15) and adhesion molecules (Icam1, Vcam1). While IRI caused a distinct up-regulation in the mRNA expression levels of Icam1, Vcam1 and, especially, in the case of Kim1 (which serves as an indicator of kidney injury [60]), ROCK-inhibition led to significantly lower expression levels of Icam1 and Kim1 while Vcam1 expression was not significantly attenuated. Since protein data might vary from gene expression analysis, PCR data might serve as indicator only. However, mRNA data might indicate that ROCK-inhibition not only affects the migration capability of leukocytes but also the capacity of the endothelium to interact with them (lower level of Icam1). ROCK is a known mediator of apoptosis as it is activated by caspases [61]. However, in our IRI model there was no difference in TUNEL staining on post-ischemic day one between HF-treated animals and controls (Figure S2, supplemental data). Since apoptosis was significantly attenuated in HF-treated animals on day four, we hypothesize that attenuation of apoptosis might rather be secondary to the inhibitory effect of HF on inflammation (as evidenced e.g. by reduced infiltration in histology and ^18^F-FDG-accumulation in the outer medulla). Since functional data implicate an improvement of kidney function as shown for example by creatinine clearance as early as day one we assume that inhibition of apoptosis is not primarily involved in the beneficial effects of ROCK-inhibition by HF. However, inhibition of apoptosis could at least in part contribute to the effects observed.

The clinical field of application of ROCK-inhibitors (e.g. Fasudil) lies within cardiovascular diseases, where mainly vasodilatory and therefore blood-pressure lowering effects of ROCK-inhibitors are contemplated manner. As tonic modulation of renal arterioles and the descending vasa recta contributes to the regulation of oxygen supply and medullary blood-flow [62], one of our hypotheses was that HF-related renal protection from IRI is related to vasodilatation resulting in improved kidney perfusion immediately after ischemia induction.

To elucidate this hypothesis, we non-invasively measured animal blood-pressures before surgery as well as renal perfusion by ^18^F-PET after surgery and calculated a modified Hilson's Perfusion index. Notably, renal perfusion did neither differ between groups at baseline measurements nor immediately after the ischemia procedure. Blood-pressure was not affected, too. Thus, in IRI acute vasodilatory effects of HF are not decisive for kidney protection. However, while renal perfusion in vehicle-treated animals was still impaired on POD4, renal perfusion of HF-treated rats had completely recovered. The reduced perfusion of vehicle-treated kidneys might be related to an increased endothelial permeability leading to interstitial edema (higher kidney weights in the NxIRCTR group) compressing the peritubular capillaries, to increased leukocyte adherence, and the extravascular accumulation of leukocytes [62]. ROCKs are central to cell-cell contacts, to cellular contraction and therefore to endothelial barrier function. Interestingly, the integrity of the endothelium is distinctly compromised in IRI, leading to extravasation of leukocytes and interstitial edema. As we observed more edema in kidneys of control animals than in HF-treated ones, we tested the endothelial barrier function/permeability *in vivo* in the first 30 minutes of the reperfusion phase. Notably, dye-accumulation was significantly reduced when ROCK-inhibition was applied indicating preserved endothelial function and integrity after IRI. This view is supported by data obtained in a model of ischemic stroke in mice. Ischemia of the middle cerebral artery induced endothelial contraction and disruption of the blood brain barrier with increased permeability that was prevented by ROCK-inhibition [63]. Since leukocyte transmigration depends on trans-endothelial migration, increased tightness of the endothelium is likely to result in reduced migration and infiltration. This decreases inflammation of the kidney. Therefore, the data suggests that improved kidney function after ROCK-inhibition was mainly mediated by affecting the inflammatory response and reducing edema compressing the peritubular capillaries. On one hand, migratory and adhesive capabilities of leukocytes were substantially reduced, either by direct effects or through the endothelium by affecting endothelial adhesion molecules and endothelial permeability. On the other hand, possible direct effects of HF on renal perfusion due to ROCK-inhibition related vasodilatation could be excluded.

We conclude from our study that ROCK-inhibition by HF significantly improves kidney function in a rat model of acute renal IRI. This was related to preserved endothelial function and lower expression of vascular adhesion molecules resulting in reduced/modulated infiltration and activity of tissue destructing Mmps, less edema and better perfusion. Therefore, ROCK-inhibition is a promising potential therapeutic target in renal IRI.

## Supporting Information

Figure S1 **Effect of ischemia-reperfusion injury and ROCK-inhibition on mRNA-expression.** Fold change in mRNA-expression compared to uninephrectomized animals (whole kidney lysates) of pro-inflammatory cytokines (Ifng, Tnf, Tnfsf15) on POD1 and POD4. As analyzed by real-time PCR, IRI did not lead to a distinct up-regulation of these gene transcripts, neither in the ischemic control group (NxIRCTR), nor in the ROCK-inhibitor treated animals (NxIRHF). Values are expressed as mean ± SEM, n = 4–6. **a** p<0.05 vs. Nx, **b** p<0.05 vs. NxIRCTR POD1 or NxIRCTR POD4, respectively.
(TIF)

Figure S2 **Effects of ROCK-inhibition on ischemia-reperfusion injury-induced apoptosis.** A) Representative TUNEL-stainings (cortex and medulla) of post-ischemic kidneys on POD1 and POD4 (NxIRCTR, NxIRHF), as well as from kidneys of healthy (CTR) and uninephrectomized animals (Nx). On POD1 there was only a slightly increased frequency of TUNEL-positive nuclei in the untreated ischemic animals (NxIRCTR) as well as in ROCK-inhibitor treated ones (NxIRHF). However, on POD 4 kidneys of NxIRCTR animals presented increased numbers of TUNEL-positive nuclei, whereas kidneys of ROCK-inhibitor-treated animals (NxIRHF) showed less TUNEL-positive stainings. B) Quantification of TUNEL-positive nuclei (#/a.u.): CTR: 5±2, Nx: 2±1; NxIRCTR POD1: 32±12, NxIRHF POD1: 35±32, NxIRCTR POD4: 239±114, NxIRHF POD4: 62±24. Values are expressed as mean ± SEM, n = 3; * p<0.05 vs. Nx.
(TIF)
