# Supplementary figures and images for: Hydroxyfasudil-Mediated Inhibition of ROCK1 and ROCK2 Improves Kidney Function in Rat Renal Acute Ischemia-Reperfusion Injury
Source: PLoS One. 2011 Oct 21;6(10):e26419. doi: 10.1371/journal.pone.0026419 (PMC3198766; doi:10.1371/journal.pone.0026419)

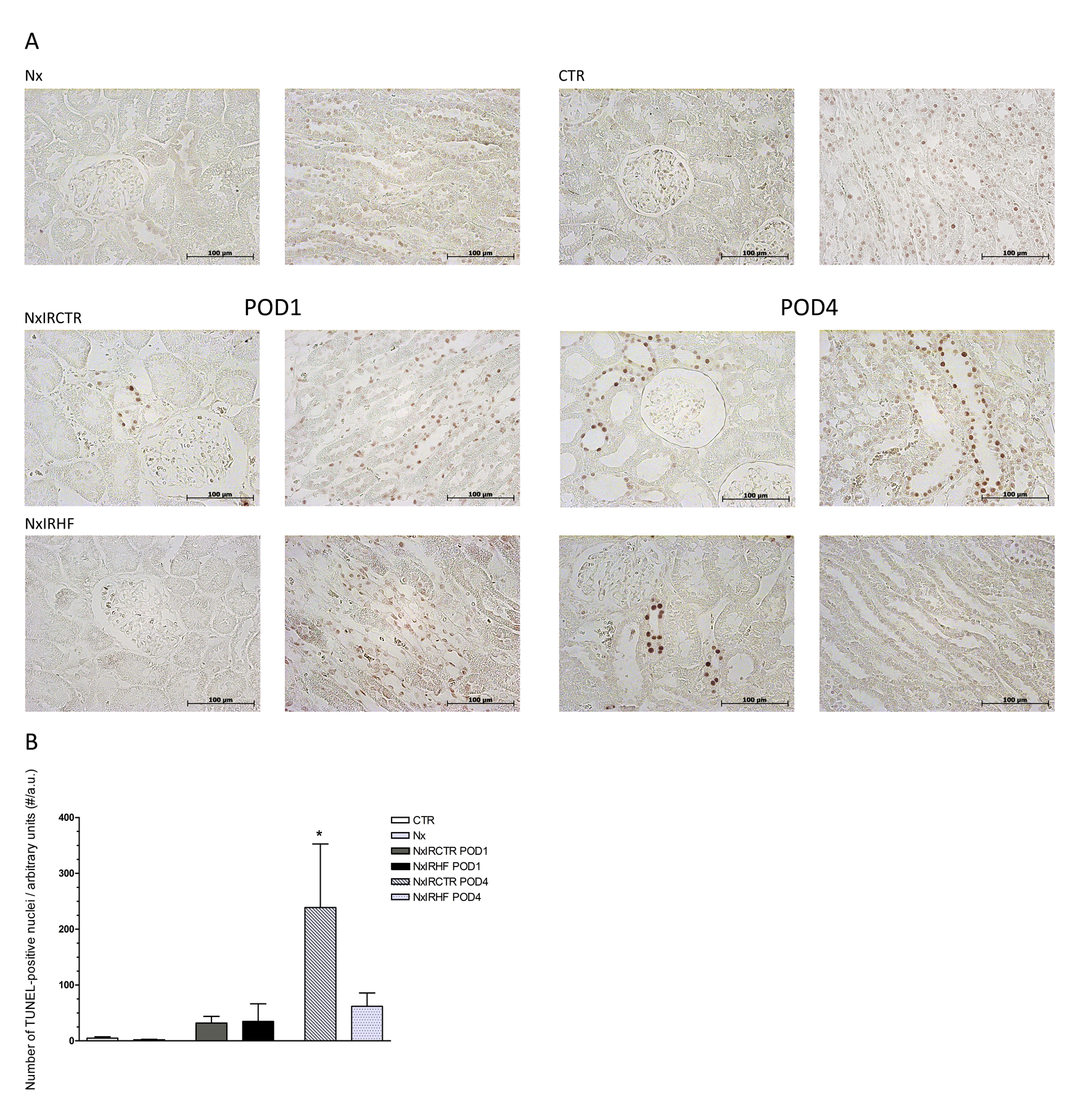

Supplement: Figure S2 — Effects of ROCK-inhibition on ischemia-reperfusion injury-induced apoptosis. A) Representative TUNEL-stainings (cortex and medulla) of post-ischemic kidneys on POD1 and POD4 (NxIRCTR, NxIRHF), as well as from kidneys of healthy (CTR) and uninephrectomized animals (Nx). On POD1 there was only a slightly increased frequency of TUNEL-positive nuclei in the untreated ischemic animals (NxIRCTR) as well as in ROCK-inhibitor treated ones (NxIRHF). However, on POD 4 kidneys of NxIRCTR animals presented increased numbers of TUNEL-positive nuclei, whereas kidneys of ROCK-inhibitor-treated animals (NxIRHF) showed less TUNEL-positive stainings. B) Quantification of TUNEL-positive nuclei (#/a.u.): CTR: 5±2, Nx: 2±1; NxIRCTR POD1: 32±12, NxIRHF POD1: 35±32, NxIRCTR POD4: 239±114, NxIRHF POD4: 62±24. Values are expressed as mean ± SEM, n = 3; * p<0.05 vs. Nx. (TIF) [file pone.0026419.s002.tif]
